# A multicenter study of body mass index in cancer patients treated with anti-PD-1/PD-L1 immune checkpoint inhibitors: when overweight becomes favorable

**DOI:** 10.1186/s40425-019-0527-y

**Published:** 2019-02-27

**Authors:** Alessio Cortellini, Melissa Bersanelli, Sebastiano Buti, Katia Cannita, Daniele Santini, Fabiana Perrone, Raffaele Giusti, Marcello Tiseo, Maria Michiara, Pietro Di Marino, Nicola Tinari, Michele De Tursi, Federica Zoratto, Enzo Veltri, Riccardo Marconcini, Francesco Malorgio, Marco Russano, Cecilia Anesi, Tea Zeppola, Marco Filetti, Paolo Marchetti, Andrea Botticelli, Gian Carlo Antonini Cappellini, Federica De Galitiis, Maria Giuseppa Vitale, Francesca Rastelli, Federica Pergolesi, Rossana Berardi, Silvia Rinaldi, Marianna Tudini, Rosa Rita Silva, Annagrazia Pireddu, Francesco Atzori, Rita Chiari, Biagio Ricciuti, Andrea De Giglio, Daniela Iacono, Alain Gelibter, Mario Alberto Occhipinti, Alessandro Parisi, Giampiero Porzio, Maria Concetta Fargnoli, Paolo Antonio Ascierto, Corrado Ficorella, Clara Natoli

**Affiliations:** 1 Medical Oncology, St. Salvatore Hospital, L’Aquila, Italy; 2 Department of Biotechnological and Applied Clinical Sciences, University of L’Aquila, L’Aquila, Italy; 3 Medical Oncology, University Hospital of Parma, Parma, Italy; 4 Department of Medicine and Surgery, University of Parma, Parma, Italy; 5 Medical Oncology, Campus Bio-Medico University, Rome, Italy; 6 Department of Clinical and Molecular Medicine, Sant’Andrea Hospital, Sapienza University of Rome, Rome, Italy; 7 Clinical Oncology Unit, S.S. Annunziata Hospital, Chieti, Italy; 8 Department of Medical, Oral & Biotechnological Sciences University G. D’Annunzio, Chieti-Pescara, Italy; 9 Medical Oncology, Santa Maria Goretti Hospital, Latina, Italy; 10 Department of Oncology, University Hospital of Pisa, Istituto Toscano Tumori, Pisa, Italy; 11 Medical Oncology, “Santo Spirito” Hospital, Pescara, Italy; 12 Istituto Dermopatico dell’Immacolata, IDI-IRCCS, Rome, Italy; 13 Medical Oncology, University Hospital of Modena, Modena, Italy; 14 Medical Oncology, Fermo Area Vasta 4, Fermo, Italy; 15 Oncology Clinic, Università Politecnica delle Marche, Ospedali Riuniti di Ancona, Ancona, Italy; 16 Medical Oncology, AV2 Fabriano ASUR Marche, Pescara, Italy; 17 Medical Oncology Unit, University Hospital of Cagliari, Cagliari, Italy; 18 Medical Oncology, Santa Maria della Misericordia Hospital, Perugia, Italy; 19 Pulmonary Oncology Unit, St. Camillo Forlanini Hospital, Rome, Italy; 20 Medical Oncology (B), Policlinico Umberto I, “Sapienza” University of Rome, Rome, Italy; 21 Dermatology, San Salvatore Hospital, L’Aquila, Italy; 22 Melanoma, Cancer Immunotherapy and Development Therapeutics Unit, Istituto Nazionale Tumori-IRCCS Fondazione “G. Pascale”, Naples, Italy; 23 Medical Oncology Unit, St. Salvatore Hospital, Department of Biotechnological and Applied Clinical Sciences, University of L’Aquila, Via Vetoio, 67100 L’Aquila, Italy

**Keywords:** BMI, Anti-PD-1/PD-L1, Overweight, Obesity, Cancer, Immunotherapy

## Abstract

**Background:**

Recent evidence suggested a potential correlation between overweight and the efficacy of immune checkpoint inhibitors (ICIs) in cancer patients.

**Patients and methods:**

We conducted a retrospective study of advanced cancer patients consecutively treated with anti-PD-1/PD-L1 inhibitors, in order to compare clinical outcomes according to baseline BMI levels as primary analysis. Based on their BMI, patients were categorized into overweight/obese (≥ 25) and non-overweight (< 25). A gender analysis was also performed, using the same binomial cut-off. Further subgroup analyses were performed categorizing patients into underweight, normal weight, overweight and obese.

**Results:**

Between September 2013 and May 2018, 976 patients were evaluated. The median age was 68 years, male/female ratio was 663/313. Primary tumors were: NSCLC (65.1%), melanoma (18.7%), renal cell carcinoma (13.8%) and others (2.4%). ECOG-PS was ≥2 in 145 patients (14.9%). PD-1/PD-L1 inhibitors were administered as first-line treatment in 26.6% of cases. Median BMI was 24.9: 492 patients (50.6%) were non-overweight, 480 patients (50.4%) were overweight/obese. 25.2% of non-overweight patients experienced irAEs of any grade, while 55.6% of overweight/obese patients (*p* < 0.0001). ORR was significantly higher in overweight/obese patients compared to non-overweight (p < 0.0001). Median follow-up was 17.2 months. Median TTF, PFS and OS were significantly longer for overweight/obese patients in univariate (p < 0.0001, for all the survival intervals) and multivariate models (*p* = 0.0009, p < 0.0001 and p < 0.0001 respectively). The significance was confirmed in both sex, except for PFS in male patients (*p* = 0.0668).

**Conclusions:**

Overweight could be considered a tumorigenic immune-dysfunction that could be effectively reversed by ICIs. BMI could be a useful predictive tool in clinical practice and a stratification factor in prospective clinical trials with ICIs.

**Electronic supplementary material:**

The online version of this article (10.1186/s40425-019-0527-y) contains supplementary material, which is available to authorized users.

## Key message

Recent evidence revealed that adipose tissue might affect the response to immune checkpoint inhibitors (ICIs) in cancer patients. In this retrospective transverse study, enrolling 976 advanced cancer patients treated with anti-PD-1/PD-L1 immunotherapy, we found a significant association between overweight (BMI ≥ 25) and improved clinical outcomes to ICIs.

## Introduction

Although the interaction between malnutrition and chronic inflammation has been widely investigated, whether this association is causative or correlative is still debated [1]. Historically, body mass index (BMI) has been considered the major surrogate of nutritional status and its correlation with clinical outcomes in advanced cancer patients has already been investigated without conclusive results [2–5].

It is now becoming clear that the nutritional assessment, which should include BMI, could be seen in a "new light" in the era of immune checkpoint inhibitors (ICIs). A large retrospective study has recently found an association between BMI and improved progression free survival (PFS) and overall survival (OS) in melanoma patients treated with either targeted therapy or immunotherapy [6]. Another study has reported that overweight sarcopenic melanoma patients treated with anti-PD1 (Programmed cell death protein 1) inhibitors experienced early acute limiting toxicity [7].

Additionally, another retrospective analysis by Richtig et al. revealed that overweight (BMI ≥ 25) melanoma patients (76 total) treated with ipilimumab had significantly higher response rate (p = 0.024) and a trend for longer OS (p = 0.056), when compared to non-overweight patients [8].

Lastly, Wang and colleagues have recently reported an improvement in terms of PFS (p = 0.003) and OS (p = 0.049) in a cohort of obese advanced cancer patients (BMI ≥ 30) treated with ICIs [9].

To further dissect this question, we conducted a large, multicentre, retrospective transverse study to evaluate clinical outcomes of patients with advanced solid tumors treated with ICIs according to baseline BMI.

## Materials and methods

### Patient eligibility

This study enrolled patients with confirmed diagnosis of measurable stage IV cancer, who consecutively underwent treatment with single agent anti-PD-1/PD-L1 as 1^st^ or subsequent line, at the medical oncology departments of 17 Italian centers (Additional file 1), between September 2013 and May 2018.

### Anthropometric measurements

Weight and height were obtained from the patient’s medical records at the time of immunotherapy initiation. BMI was calculated using the formula of weight/height^2^ (kilograms per square meter) and classified according to the World Health Organization (WHO) categories: underweight, BMI < 18.5; normal, 18.5 ≤ BMI ≤ 24.9; overweight, 25 ≤ BMI ≤ 29.9; obesity, BMI ≥ 30. For the study purpose, the binomial cut-off for BMI </≥ 25 was used, and patients were categorized into non-overweight (< 25) and overweight/obese (≥ 25) for the final analysis. Underweight patients were included in the non-overweight group.

### Study design

We conducted a “real-life”, multicenter, retrospective observational study aimed at comparing the clinical outcomes of cancer patients treated with ICIs according to baseline BMI levels.

Primary outcomes measures were: objective response rate (ORR), time to treatment failure (TTF), PFS and OS. ORR was defined as the proportion of patients experiencing an objective response (either complete response or partial response) as best response to immunotherapy. TTF was defined as the time from treatment’s start to discontinuation for any reason. Progression-free survival (PFS) was defined as the time from the start of immunotherapy to the date of disease progression or death, whichever occurred first. Patients who were alive without disease progression were censored on the date of their last disease assessment. Overall survival (OS) was defined as the time from the start of immunotherapy to death. Patients who were still alive were censored at the date of last contact. Patients were treated according to the tumor type indication with pembrolizumab, nivolumab or atezolizumab with standard doses and schedules.

In order to weighing the possible prognostic influence of obesity (30 BMI) and malnutrition (or cachexia), two subgroup analysis (according to each BMI categories) were performed. In the first one, overweight (25-30 BMI) and obese (≥ 30 BMI) patients were respectively compared to non-overweight (< 25) patients, in the second one overweight (25-30 BMI) and obese (≥ 30 BMI) patients were respectively compared to normal weight patients (18.5-25 BMI).

A subgroup analysis comparing clinical outcomes in males and females patients, using the binomial cut-off (BMI </≥ 25) was also conducted as secondary analysis.

The following covariates were considered for the multivariate analyses: primary tumor (NSCLC, melanoma, kidney and others), sex (male *vs* female), Eastern Cooperative Oncology Group Performance Status (ECOG-PS) (0-1 *vs.* ≥ 2), age (< 70 *vs.* ≥ 70 years old) [10–13], number of metastatic sites (≤ 2 *vs.* > 2) and treatment line (first *vs* non-first). As in some indications the anti-PD-1/PD-L1 agents dosages had been weight-based, weight was used as a continuous covariate in all the analyses, considering the possible dose-depending confounding effect on the clinical outcomes.

Immune-related AEs (irAEs) were graduated according to the Common Toxicity Criteria for Adverse Events (CTCAE; version 4.0) and cumulatively reported. Immune-related AEs were categorized on the basis of the organ/system involved as follows: endocrine irAEs (including thyroid disorders), gastro-intestinal (GI) irAEs (excluding pancreatitis), skin irAEs, pneumological irAEs, hepatic irAEs, rheumatologic irAEs and others irAEs (including neuro-muscolar, pancreatitis, fever, asthenia and anorexia). The safety analysis was performed for irAEs of any grade and for G3/G4 irAEs.

To determine ORR and PFS, scans were reviewed by a dedicated thoracic oncologist at each Institution using Response Evaluation Criteria In Solid Tumors (RECIST) version 1.1. [14]. χ2 was used to compare ORR and incidence of irAEs among subgroups [15]. In the multivariate analysis, logistic regression was used to evaluate the role of parameters proven to be significant at the univariate analysis of ORR [16]. Median TTF, median PFS, and median OS were evaluated using the Kaplan-Meier method [17]. Median follow-up was calculated according to the reverse Kaplan-Meier method [18]. Cox proportional hazards model [19] was used to evaluate predictor variables in univariate and multivariate analysis for TTF, PFS and OS. The data cut-off was October 29^th^, 2018. All statistical analyses were performed using MedCalc Statistical Software version 18.6 (MedCalc Software bvba, Ostend, Belgium; http://www.medcalc.org; 2018).

## Results

### Patient characteristics

Nine hundred and seventy-six, consecutive advanced cancer patients were evaluated. Patient characteristics are summarized in Table 1. The median age was 68 years (range: 24 – 92), male/female ratio was 663/313. Primary tumors were: NSCLC (635 patients), melanoma (183 patients), renal cell carcinoma (135 patients) and others (23 patients). ECOG-PS was 0/1 in 831 patients (85.1%), and ≥ 2 in 145 patients (14.9%); 467 patients (47.9%) had ≤ 2 metastatic sites while 509 (52.1%) had more than 2 metastatic sites. PD-1/PD-L1 inhibitors were administered as first-line treatment in 260 patients (26.6%). Median weight was 71 Kg, median BMI was 24.9; according to WHO classification 40 patients (4.1%) were defined as underweight, 452 patients (46.3%) as having a normal weight, 377 patients (38.6%) as overweight and 107 patients (11%) as obese. For the study purpose, 492 patients were considered as non-overweight (50.4%) and 484 patients were categorized as overweight/obese (49.6%) according to a BMI cut-off of 25 (<25 *vs.* ≥25).

**Table 1:** Patients’ characteristics

	N° (%)
	976
AGE, (years)	
Median	68
Range	24–92
Elderly (≥ 70)	445 (45.6)
SEX	
Male	663 (67.9)
Female	313 (32.1)
ECOG PS	
0–1	831 (85.1)
≥ 2	145 (14.9)
Primary Tumor	
NSCLC	635 (65.1)
Melanoma	183 (18.7)
Renal cell carcinoma	135 (13.8)
Others	23 (2.4)
No. of metastatic sites	
≤ 2	467 (47.9)
> 2	509 (52.1)
Type of anti-PD-1/PD-L1 agent	
Pembrolizumab	235 (24.1)
Nivolumab	706 (72.3)
Atezolizumab	35 (3.6)
Treatment line of Immunotherapy	
First	260 (26.6)
Non-First	716 (73.4)
Weight (Kg)	
Median	71
Range	35–139
BMI (kg/m^2^)	
Median (range)	24.9 (13.5–46.6)
Underweight (BMI ≤ 18.5), n°(%)	40 (4.1)
Normal weight (BMI 18.5 < BMI ≤ 24.9), n°(%)	452 (46.3)
Overweight (25 < BMI ≤ 29.9), n°(%)	377 (38.6)
Obese (BMI ≥ 30), n° (%)	107 (11)

Among male patients median age was 69 years, median weight was 72 Kg (range: 35 – 139) and median BMI was 24.8 (range: 14 – 46.6). Among female patients median age was 67, median weight was 70 Kg (range: 40 – 130) and median BMI was 25.4 (range: 13.6 – 46.1).

### Safety analysis

In the entire cohort, 393 patients (40.3%) experienced irAEs of any grade. Sixty-three patients (6.5%) experienced G3/G4 irAEs. Overweight/obese patients were significantly more likely to experience any grade irAEs compared to non-overweight patients (55.6% *vs.* 25.2%, p < 0.0001). However, no difference in the rate of G3/G4 irAEs was observed between Overweight/obese patients and non-overweight patients (7.6 *vs.* 5.3%, p = 0.1338). The safety profile of ICIs according to BMI is summarized in Additional file 2.

### Activity analysis

Univariate and multivariate analyses for ORR are detailed in Additional file 3. Among 910 patients evaluable for activity, 283 patients had a response to ICIs (ORR: 31.1%). Overweight/obese patients had a significantly higher ORR compared non-overweight patients (41.3% *vs.* 20.9%, p < 0.0001). Similarly, we found a significantly higher ORR among patients who experienced at least 1 irAE compared to those without irAEs (45.1% *vs.* 21.1%, p < 0.0001). Both BMI (overweight/obese *vs.* non-overweight) and the development of irAEs of any grade, were independently associate with higher ORR in the multivariate analysis (p = 0.0239 and p < 0.0001, respectively).

### Efficacy analysis

At median follow-up of 17.2 months, median TTF was 5.9 months (95% CI: 5.3 – 6.7; 681 events), median PFS was 6.5 months (95% CI: 6.1 – 7.1; 644 events) and median OS was 13.4 months (95% CI: 11.0 – 16.5; 488 censored patients) in the entire cohort.

**Fig. 1:**
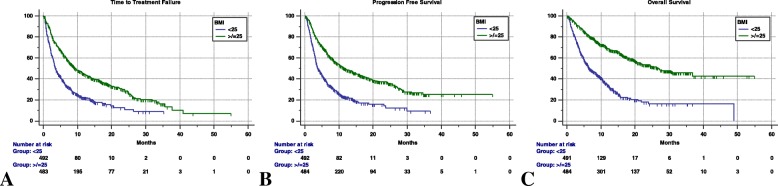
Kaplan-Meier survival curves according to binomial BMI levels (cut-off 25). (**a**) Time to Treatment Failure. BMI < 25: 3.6 months (95% CI: 3.2–4.1); BMI ≥ 25: 9.3 months (95%CI: 8.1–11.6). (**b**) Progression Free Survival. BMI < 25: 3.7 months (95% CI: 3.2–4.1); BMI ≥ 25: 11.7 months (95% CI: 9.4–15). (C) Overall Survival. BMI < 25: 6.6 months (95% CI: 5.8–8.5); BMI ≥ 25: 26.6 months (95% CI: 21.4–36.8)

When these outcomes where analyzed according to BMI, we found that median TTF was significantly longer in overweight/obese patients compared to non-overweight patients (9.3 [95% CI: 8.1 – 11.6; 318 events] *vs.* 3.6 months [95% CI: 3.2 – 4.1; 363 events]; HR= 0.51 [95% CI: 0.44 – 0.60], p < 0.0001) (Fig. 1a). Similarly, median PFS was significantly improved in the overweight/obese group compared to the non-overweight group (11.7 months [95% CI: 9.4 – 15; 286 events] *vs*. 3.7 months [95% CI: 3.2 – 4.1; 358 events]; HR= 0.46 [95%CI: 0.39 – 0.54], p < 0.0001) (Fig. 1b). Consistently we also found a significantly prolonged median OS among overweight/obese patients compared to non-overweight patients (26.6 months [95% CI: 21.4 – 36.8; 286 censored patients] *vs.* 6.6 months [95% CI: 5.8 – 8.5; 182 censored patients]; HR= 0.33 [95%CI: 0.28 – 0.41], p < 0.0001) (Fig. 1c).

After adjusting for PS, treatment line, n° of metastatic sites, gender, primary tumor subtype and development of irAEs, a BMI of ≥25 retained a significant association with a longer TTF (p = 0.0009), PFS (p < 0.0001) and OS (p < 0.0001) in multivariate models (Table 2, Table 3, Table 4)

**Table 2:** Cox proportional-hazards regression: univariate and multivariate analyses of Time to Treatment Failure

	Time to Treatment Failure	
	Univariate Analysis	Multivariate Analysis
VARIABLE (Comparator)	HR (95% CI); *p - value*	HR (95% CI); *p - value*
BMI ≥ 25 vs < 25	0.51 (0.44–0.60); *p < 0.0001*	0.67 (0.53–0.85); *p = 0.0009*
Weight^a^	0.98 (0.97–0.99); *p < 0.0001*	0.99 (0.98–1.01); *p = 0.8422*
irAEs of any grade Yes vs No	0.57 (0.48–0.66); *p < 0.0001*	0.79 (0.65–0.97); *p = 0.0295*
Primary Tumor (NSCLC)		
Melanoma Kidney Others	0.62 (0.50–0.76); *p < 0.0001* 0.73 (0.59–0.92); *p = 0.0077* 1.15 (0.71–1.87); *p = 0.5560*	0.79 (0.64–1.01); *p = 0.0517* 0.71 (0.56–0.88); *p = 0.0025* 0.78 (0.48–1.28); *p = 0.3389*
Sex Male vs Female	1.22 (1.04–1.43); *p = 0.0147*	1.10 (0.93–1.30); *p = 0.2607*
Age Elderly vs Non-elderly	1.04 (0.90–1.21); *p = 0.5366*	–
Treatment line Non-first vs First	1.36 (1.13–1.64); *p = 0.0008*	1.51 (1.25–1.81); *p < 0.0001*
N° of metastatic sites > 2 vs ≤ 2	1.54 (1.34–1.77); *p < 0.0001*	1.52 (1.30–1.77); *p < 0.0001*
ECOG PS ≥2 vs 0–1	2.86 (2.36–3.48); *p < 0.0001*	2.35 (1.92–2.88); *p < 0.0001*

^a^Weight was used as a continuous variable

**Table 3:** Cox proportional-hazards regression: univariate and multivariate analyses of Progression Free Survival

	Progression Free Survival	
	Univariate Analysis	Multivariate Analysis
VARIABLE (Comparator)	HR (95% CI); *p - value*	HR (95% CI); *p - value*
BMI ≥ 25 vs < 25	0.46 (0.39–0.54); *p < 0.0001*	0.71 (0.56–0.90); *p < 0.0001*
Weight^a^	0.97 (0.96–0.98); *p < 0.0001*	0.99 (0.98–1.01); *p = 0.1580*
irAEs of any grade Yes vs No	0.48 (0.41–0.57); *p < 0.0001*	0.67 (0.54–0.83); *p = 0.0002*
Primary Tumor (NSCLC)		
Melanoma	0.52 (0.42–0.66); *p < 0.0001*	0.67 (0.53–0.85); *p = 0.0008*
Kidney	0.72 (0.58–0.91); *p = 0.0062*	0.67 (0.53–0.84); *p = 0.0008*
Others	1.08 (0.65–1.78); *p = 0.7556*	0.69 (0.41–1.15); *p = 0.1533*
Sex Male vs Female	1.20 (1.01–1.42); *p = 0.0314*	1.03 (0.86–1.22); *p = 0.7252*
Age Elderly vs Non-elderly	0.96 (0.82–1.12); *p = 0.6394*	–
Treatment line Non-first vs First	1.62 (1.33–1.96); *p < 0.0001*	1.61 (1.32–1.93); *p < 0.0001*
N° of metastatic sites > 2 vs ≤ 2	1.46 (1.27–1.68); *p < 0.0001*	1.42 (1.21–1.67); *p < 0.0001*
ECOG PS ≥2 vs 0–1	2.60 (2.13–3.17); *p < 0.0001*	2.06 (1.67–2.52); *p < 0.0001*

^a^Weight was used as a continuous variable

**Table 4:** Cox proportional-hazards regression: univariate and multivariate analyses of Overall Survival

	Overall Survival	
	Univariate Analysis	Multivariate Analysis
VARIABLE (Comparator)	HR (95% CI); *p – value*	HR (95% CI); *p - value*
BMI ≥ 25 vs < 25	0.33 (0.28–0.41); *p < 0.0001*	0.49 (0.38–0.64); *p < 0.0001*
Weight^a^	0.97 (0.96–0.97); *p < 0.0001*	0.99 (0.99–1.01); *p = 0.1884*
irAEs of any grade Yes vs No	0.45 (0.37–0.54); *p < 0.0001*	0.82 (0.65–1.04); *p = 0.1085*
Primary Tumor (NSCLC)		
Melanoma	0.49 (0.38–0.64); *p < 0.0001*	0.67 (0.51–0.87); *p = 0.0036*
Kidney	0.56 (0.42–0.74); *p = 0.0001*	0.61 (0.45–0.80); *p = 0.0005*
Others	1.11 (0.62–1.96); *p = 0.7337*	0.71 (0.40–1.28); *p = 0.2632*
Sex Male vs Female	1.50 (1.23–1.83); *p < 0.0001*	1.33 (1.09–1.63); *p = 0.0044*
Age Elderly vs Non-elderly	1.11 (0.93–1.32); *p = 0.2401*	–
Treatment line Non-first vs First	1.58 (1.26–1.97); *p = 0.0001*	1.42 (1.15–1.77); *p = 0.0012*
N° of metastatic sites > 2 vs ≤ 2	1.52 (1.29–1.78); *p < 0.0001*	1.41 (1.17–1.69); *p = 0.0002*
ECOG PS ≥2 vs 0–1	2.07 (1.87–2.29); *p < 0.0001*	2.59 (2.09–3.21); *p < 0.0001*

^a^Weight was used as a continuous variable

### Subgroup analyses

Table 5 reports the univariate and multivariate gender analyses for TTF, PFS and OS of male patients (Table 5A) and female patients (Table 5B). As shown overweight/obese male patients had significantly longer TTF (p = 0.0330) and OS (p = 0.0013), but not PFS (p = 0.0668), when compared with non-overweight patients, while overweight/obese female patients had significantly longer TTF (p = 0.0037), PFS (p = 0.0132) and OS (p < 0.0001), when compared to non-overweight patients.

**Table 5:** Cox proportional-hazards regression: univariate and multivariate analyses

A	Univariate Analysis	Multivariate Analysis
VARIABLE	HR (95% CI); *p - value*	HR (95% CI); *p - value*
	Time to Treatment Failure	
BMI ≥ 25 vs < 25	0.54 (0.45–0.66); *p < 0.0001*	0.74 (0.56–0.97); *p = 0.0330*
	Progression Free Survival	
BMI ≥ 25 vs < 25	0.49 (0.40–0.59); *p < 0.0001*	0.77 (0.58–1.01); *p = 0.0668*
	Overall Survival	
BMI ≥ 25 vs < 25	0.38 (0.31–0.48); *p < 0.0001*	0.59 (0.43–0.81); *p = 0.0013*
B	Univariate Analysis	Multiavariate Analysis
VARIABLE	HR (95% CI); *p - value*	HR (95% CI); *p - value*
	Time to Treatment Failure	
BMI ≥ 25 vs < 25	0.45 (0.35–0.61); *p < 0.0001*	0.51 (0.32–0.80); *p = 0.0037*
	Progression Free Survival	
BMI ≥ 25 vs < 25	0.41 (0.31–0.56); *p < 0.0001*	0.56 (0.35–0.88); *p = 0.0132*
	Overall Survival	
BMI ≥ 25 vs < 25	0.25 (0.17–0.36); *p < 0.0001*	0.27 (0.15–0.48); *p < 0.0001*

(A) male patients (B) female patients. The used covariates (not shown) were: weight (continuous), irAEs of any grade, primary tumors, line of treatment, ECOG-PS, number of metastatic sites

**Fig. 2:**
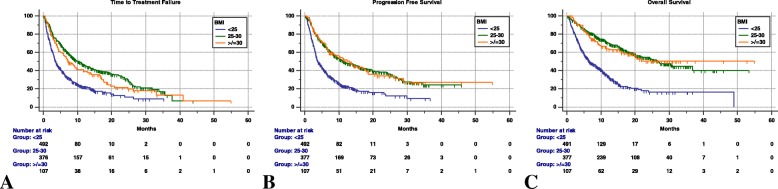
Kaplan-Meier survival curves according to BMI levels (non-overweight BMI < 25, overweight BMI 25–30, obese BMI ≥ 30). (**a**) Time to Treatment Failure. BMI < 25: 3.6 months (95% CI: 3.2–4.1); BMI 25–30: 10.3 months (95%CI: 8.2–4.1); BMI ≥ 30: 7.3 months (95%CI: 5.5–11.7). (**b**) Progression Free Survival. BMI < 25: 3.7 months (95% CI: 3.2–4.1); BMI 25–30: 11.2 months (95%CI: 9.1–15.6); BMI ≥ 30: 12.9 months (95%CI: 7.1–18). (**c**) Overall Survival. BMI < 25: 6.6 months (95% CI: 5.8–8.5); BMI 25–30: 26.6 months (95%CI: 21.4–36.8); BMI ≥ 30: not reached

Median TTF was not significantly different between overweight and obese patients (10.3 months [95%CI: 8.2 – 4.1; 238 events] *vs.* 7.3 [95%CI: 5.5 – 11.7; 80 events], HR=1.23 [95%CI: 0.95 – 1.58], p = 0.1087). Similarly, we found no significant differences in median PFS (11.2 months [95%CI: 9.1 – 15.6; 223 events patients] *vs.* 12.9 months [95%CI: 7.1 – 18; 63 events], HR=0.99 [95%CI: 0.75 – 1.31], p = 0.9798) and median OS (26.6 months [95%CI: 21.4 – 36.8; 223 censored patients] *vs.* not reached [63 censored patients], HR=1.04 [95%CI: 0.75 – 1.46], p = 0.7767) between overweight and obese patients. Table 6 reports the univariate and multivariate analyses of TTF, PFS and OS, comparing overweight (non-obese) patients and obese patient with non-overweight patients. Figure 2 reports the Kaplan-Meier survival curves of obese, overweight and non-overweight patients.

**Table 6:** Cox proportional-hazards regression: univariate and multivariate analyses according to non-overweight (< 25), overweight (25-30) and obese (≥ 30) BMI levels

	Univariate Analysis	Multivariate Analysis
VARIABLE (Comparator)	HR (95% CI); *p - value*	HR (95% CI); *p - value*
BMI (< 25)	Time to Treatment Failure	
25–30 ≥ 30	0.49 (0.41–0.58); *p < 0.0001* 0.60 (0.47–0.77); *p = 0.0001*	0.67 (0.53–0.84); *p = 0.0008* 0.86 (0.61–1.24); *p = 0.4414*
BMI (< 25)	Progression Free Survival	
25–30 ≥ 30	0.46 (0.39–0.55); *p < 0.0001* 0.46 (0.35–0.61); *p < 0.0001*	0.71 (0.56–0.89); *p = 0.0044* 0.80 (0.55–1.17); *p = 0.2669*
BMI (< 25)	Overall Survival	
25–30 ≥ 30	0.33 (0.27–0.41); *p < 0.0001* 0.34 (0.25–0.48); *p < 0.0001*	0.49 (0.37–0.64); *p < 0.0001* 0.61 (0.39–0.94); *p = 0.0258*

The used covariates (not shown) were: weight (continuous), irAEs of any grade, primary tumors, sex, line of treatment, ECOG-PS, number of metastatic sites

**Fig. 3:**
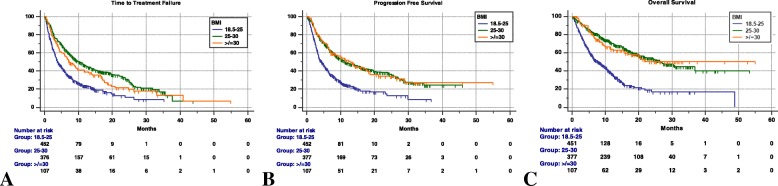
Kaplan-Meier survival curves according to BMI levels (normal weight BMI 18.5–25, overweight BMI 25–30, obese BMI ≥ 30). (**a**) Time to Treatment Failure. BMI 18.5–25: 3.9 months (95% CI: 3.4–5.0); BMI 25–30: 10.3 months (95%CI: 8.2–4.1); BMI ≥ 30: 7.3 months (95%CI: 5.5–11.7). (**b**) Progression Free Survival. BMI 18.5–25: 4.4 months (95% CI: 3.6–5.3); BMI 25–30: 11.2 months (95%CI: 9.1–15.6); BMI ≥ 30: 12.9 months (95%CI: 7.1–18). (**c**) Overall Survival. BMI 18.5–25: 7.9 months (95% CI: 6.4–9.8); BMI 25–30: 26.6 months (95%CI: 21.4–36.8); BMI ≥ 30: not reached

When we analyzed the clinical outcomes of normal weight *vs.* underweight patients, we found a significantly longer median TTF (3.9 months [95%CI: 3.4 – 5.0; 327 events] *vs.* 1.8 [95%CI: 1.7 – 2.9; 36 events], HR= 0.51 [95%CI: 0.35 – 0.71], p = 0.0001 and median PFS (4.4 months [95%CI: 3.6 – 5.3; 322 events] *vs.* 1.9 months [95%CI: 1.7 – 2.9; 36 events] HR= 0.45; 95%CI: 0.32 – 0.64], p < 0.0001) in normal weight patients compared with underweight patients. We also found a significant prolonged median OS among normal weight compared to underweight patients (7.9 months [95%CI: 6.4 – 9.8; 178 censored patients] *vs.* 2.8 months [95%CI: 1.8 – 3.6; 4 censored patients], HR= 0.33 [95%CI: 0.23 – 0.48], p < 0.0001). Table 7 reports the univariate and multivariate analyses of TTF, PFS and OS, comparing overweight (non-obese) patients and obese patients with normal weight patients. Figure 3 reports the Kaplan-Meier survival curves of obese, overweight and normal weight patients.

**Table 7:** Cox proportional-hazards regression: univariate and multivariate analyses according to normal weight (18.5-25), overweight (25-30) and obese (≥ 30) BMI levels

	Univariate Analysis	Multivariate Analysis
VARIABLE (Comparator)	HR (95% CI); *p - value*	HR (95% CI); *p - value*
BMI (18.5–25)	Time to Treatment Failure	
25–30 ≥ 30	0.51 (0.43–0.61); *p < 0.0001* 0.63 (0.49–0.81); *p = 0.0003*	0.65 (0.51–0.82); *p = 0.0004* 0.79 (0.55–1.15); *p = 0.2300*
BMI (18.5–25)	Progression Free Survival	
25–30 ≥ 30	0.49 (0.41–0.58); *p < 0.0001* 0.48 (0.37–0.64); *p < 0.0001*	0.68 (0.53–0.87); *p = 0.0016* 0.72 (0.49–1.06); *p = 0.0991*
BMI (18.5–25)	Overall Survival	
25–30 ≥ 30	0.35 (0.29–0.43); *p < 0.0001* 0.37 (0.27–0.51); *p < 0.0001*	0.46 (0.35–0.61); *p < 0.0001* 0.50 (0.32–0.79); *p = 0.0029*

The used covariates (not shown) were: weight (continuous), irAEs of any grade, primary tumors, sex, line of treatment, ECOG-PS, number of metastatic sites

## Discussion

In this study we demonstrated that patients with a BMI ≥ 25 experienced a better clinical outcome compared to those with a BMI < 25. Recently, the association between BMI and OS of metastatic renal cell carcinoma patients, has been reported regardless of the use of anti-PD-1/PD-L1 therapy [4, 20]. However, in our study we found a strong correlation between overweight and improved clinical outcomes with anti-PD-1/PD-L1.

Some authors have already speculated about the negative impact of body composition alteration on immune cells activity [21]. Interestingly, it has been increasingly recognized that white adipose tissue, which is the most related to the fattening process [22], is also involved in the induction and/or coordination of host defenses, being a source of cytokines and chemokines [23]. In fact, adipose tissue modulates the Th1/Th2 balance, decreases the activation of Treg through adiponectin, increases pro-inflammatory macrophages, activates T-cells with the binding between LIGHT-HVEM (herpesvirus entry mediator) and increases the inflammatory status through CD40 pathway [24–26].

Moreover, a recent preclinical study revealed that white adipose tissue might also play a role in immune homeostasis [27]. In this study, white adipose tissue of mice was reported to accumulate pathogen-specific memory T-cells after a microbial infection, including tissue-resident cells expressing a distinct metabolic profile. Intriguingly, these data support the hypothesis that adipose tissue can act as a reservoir of tissue-specific memory T-cells, which can undergo a rapid response to reactivation against exogenous stimuli. This evidence raises an interesting question, can these adipose tissue-specific T-cells be promptly reactivated against cancer-specific antigens as they do against microbial antigens?

In a recent meta-analysis of patients with immune-mediated inflammatory diseases treated with anti-TNF (tumor necrosis factor), the authors reported a trend towards a lower response rate to treatment among overweight patients [28]. This is likely to reflect the reduced responsivity of T-cells of obese individuals, which has also been confirmed in preclinical models showing a significant increase in dysfunctional exhausted T-cells in obese mice [9]. Nevertheless, such inflamed and immune-exhausted status may be more likely susceptible to the immune checkpoint blockade. In support of this, in preclinical models, T-cell dysfunction in obese mice was proven to be partly mediated by the PD-1 axis and driven by leptin, strengthening the already known correlation between JAK/STAT pathway and immune checkpoint inhibition [9, 29].

 Importantly, in our study we also found a significantly higher incidence of irAEs of any grade among overweight/obese patients. In light of the emerging association between the development of irAEs and improved clinical outcomes with ICIs across different tumor types, our findings are not unexpected [30–35]. In our cohort, the development of irAEs of any grade was independently associated with improved clinical outcomes along with a BMI ≥25 in multivariate analyses.

The analysis performed by separating overweight and obese patients, demonstrated that a linear relationship between BMI and positive outcomes cannot be assumed. Even though we found no statistically significant differences in TTF, PFS and OS between overweight and obese patients, when separately comparing obese patients to non-overweight patients (Table 6), we observed the loss of significance regarding TTF and PFS, while not regarding OS. This result is of particular interest, considering the possibility of a negative impact on survival of obese patients due to cardiovascular and metabolic complications of obesity itself. Noteworthy, the HRs are concordantly lower for overweight (non-obese) patients in each survival analysis, compared to obese patients, thus supporting the hypothesis that the prognostic weight of obesity, could have partially influenced the final results.

On the other hand, despite the small sample size (4.1% of the entire population), underweight patients had significantly shorter TTF, PFS and OS, when compared to normal weight patients, confirming that malnutrition (and cachexia) is an independent negative prognostic factor. Nonetheless, when we compared obese and overweight patients (Table 7) with normal weight patients we observed significantly improved clinical outcomes favouring the overweight group, which suggests that overweightness has a direct impact on the efficacy of ICIs.

In our study we also carried a gender-based analysis. Previously, it has been reported that female patients tend to have lower benefit from ICI compared to males [36, 37]. However, whether the gender plays a key role in determining the clinical outcome to immunotherapy is still in need of further investigation. In our study, we found that overweight female patients derived a greater clinical benefit form immunotherapy as compared to the male counterpart (Table 5). However, it should be highlighted that overweightness was associated with improved outcomes in both males and females in the multivariate analysis. This led us to speculate that in our population the predictive role of BMI was to be stronger than the predictive role of gender.

Certainly, the relationship between sex, adipose tissue and immunity is complex and ambiguous. Sex-hormones, in particular estrogens, could affect adipose tissue functions [38], but in some respects their influence on the immune systems does not seem unidirectional [39]. Furthermore, the median age of female patients in our study was 67, indicating a prevalence of postmenopausal patients. In this specific population the adipose tissue becomes a major source of circulating estrogens [40].

Another way to explain how BMI might affect sex hormones levels and the immune response, is through diet regimens which underpin the weight gain. Indeed, gut microbiota may be influenced by the different "modifying pressures" of various diet types. Interestingly, males and females have recently been reported to have gender-specific differences in their immune system and gut microbiota composition. Whether these differences in gut microbiota composition might impact the efficacy or the safety profile of immunotherapy is subject of intense research and is expected to provide us further insight in the optimal management of our patients [41–43].

Our study is certainly flawed by several caveats, including the retrospective design with the risk of selection and data collection biases, the heterogeneity of the analyzed population, the lack of a centralized imaging review for response assessment and the lack of data about patient comorbidities. In addition, the lack of control group of patients who did not received ICIs further limit the power of our analysis. On the other hand, a unique strength of our study is that we evaluated the predictive role of baseline assessment of BMI in a “real life” population of individuals candidate to receive ICIs.

## Conclusion

In this study we demonstrated that patients with a BMI ≥ 25 experienced better clinical outcomes with anti-PD-1/PD-L1 agents, compared to those with a BMI < 25. Our results suggest that BMI could be a useful predictive tool in clinical practice as well as a reliable stratification variable for prospective clinical trials with ICIs.

## Additional files

Additional file 1: List of oncological institutions of the study. (DOCX 15 kb)

Additional file 2: Immune-related adverse events of any grade and G3/G4 immune-related adverse events. (DOCX 15 kb)

Additional file 3: Univariate and multivariate analyses with logistic regression of Objective Response Rate. (DOC 49 kb)
