# Supplementary material for: A multicenter study of body mass index in cancer patients treated with anti-PD-1/PD-L1 immune checkpoint inhibitors: when overweight becomes favorable
Source: J Immunother Cancer. 2019 Feb 27;7:57. doi: 10.1186/s40425-019-0527-y (PMC6391761; doi:10.1186/s40425-019-0527-y)
Supplement: Supplementary file 1 — List of oncological institutions of the study. (DOCX 15 kb) [file 40425_2019_527_MOESM1_ESM.docx]

| **Institution** | **Department** |
| --- | --- |
| St. Salvatore Hospital, University of L’Aquila, L’Aquila | Medical Oncology Unit |
| SS Annunziata Hospital, Chieti | Medical Oncology Unit |
| University Hospital of Parma, Parma | Medical Oncology Unit |
| St. Camillo Forlanini Hospital, Rome | Pulmonary Oncology Unit |
| University Hospital of Cagliari, Cagliari | Medical Oncology Unit |
| S Maria Goretti Hospital, Latina | Medical Oncology Unit |
| St. Andrea Hospital, Rome | Medical Oncology Unit |
| Campus Bio-Medico University, Rome | Medical Oncology Unit |
| Policlinico Umberto I, Rome | Medical Oncology Unit |
| “Ospedali Riuniti” Hospital, Ancona | Medical Oncology Unit |
| St. Maria della Misericordia Hospital, Perugia | Medical Oncology Unit |
| Hospital of Fabriano, Fabriano | Medical Oncology Unit |
| SS Spirito Hospital, Pescara | Medical Oncology Unit |
| Hospital of Fermo, Fermo | Medical Oncology Unit |
| St. Chiara Hospital, Pisa | Medical Oncology Unit |
| University Hospital of Modena | Medical Oncology Unit |
| Istituto Dermopatico dell’ Immacolata, IRCCS, Rome | Medical Oncology Unit |
