# Supplementary material for: A multicenter study of body mass index in cancer patients treated with anti-PD-1/PD-L1 immune checkpoint inhibitors: when overweight becomes favorable
Source: J Immunother Cancer. 2019 Feb 27;7:57. doi: 10.1186/s40425-019-0527-y (PMC6391761; doi:10.1186/s40425-019-0527-y)
Supplement: Supplementary file 2 — Immune-related adverse events of any grade and G3/G4 immune-related adverse events. (DOCX 15 kb) [file 40425_2019_527_MOESM2_ESM.docx]

|  | **irAEs of any grade** | **G3/G4 irAEs** |
| --- | --- | --- |
| **Patients** | **393** | **63** |
| **Endocrine** | 138 (35.1%) | 8 (12.7%) |
| **Gastrointestinal** | 69 (17.5%) | 17 (26.9%) |
| **Skin** | 116 (29.5%) | 10 (15.8%) |
| **Pneumological** | 31 (7.9%) | 7 (11.1%) |
| **Haepatic** | 28 (7.1%) | 13 (20.6%) |
| **Rheumatologic** | 19 (4.8%) | 1 (1.6%) |
| **Others** | 57 (14.5%) | 11 (17.5%) |
