# Supplementary material for: A multicenter study of body mass index in cancer patients treated with anti-PD-1/PD-L1 immune checkpoint inhibitors: when overweight becomes favorable
Source: J Immunother Cancer. 2019 Feb 27;7:57. doi: 10.1186/s40425-019-0527-y (PMC6391761; doi:10.1186/s40425-019-0527-y)
Supplement: Supplementary file 3 — Univariate and multivariate analyses with logistic regression of Objective Response Rate. (DOC 49 kb) [file 40425_2019_527_MOESM3_ESM.doc]

|  | **UNIVARIATE ANALYSIS** | | | **MULTIVARIATE ANALYSIS** | | |
| --- | --- | --- | --- | --- | --- | --- |
| **Variable (comparator)** | **Response/ Ratio** | **ORR (95% CI)** | ***p - value*** | **Coeff.** | **St. Err.** | ***p - value*** |
| **Overall** | 283/910 | 31.1 (27.5–34.9) | *-* | - | - | *-* |
| **BMI**  ≥ 25  < 25 | 188/455  95/455 | 41.3 (35.6–47.6)  20.9 (16.8–25.5) | *< 0.0001* | -0.4449 | 0.1969 | *0.0239* |
| **irAEs of any grade**  Yes  No | 171/379  112/531 | 45.1 (38.6–52.4)  21.1 (17.3–25.3) | *< 0.0001* | -0.8442 | 0.1924 | *<0.0001* |
| **Primary Tumor**  (NSCLC)  Melanoma  Kidney  Others | 171/592  76/177  30/125  6/16 | 28.9 (24.7-33.5)  42.9 (33.8-53.7)  24.0 (16.1-34.2)  37.5 (13.7-81.6) | *0.0010* | -0.2069  0.3436  -0.9880 | 0.2101  0.2429  0.5408 | *0.3248*  *0.1573*  *0.0677* |
| **Sex**  Male  Female | 191/614  92/296 | 31.1 (26.8–35.8)  31.1 (25.1-38.1) | *0.9936* | - | - | *-* |
| **Age**  Elderly  Non-elderly | 130/406  153/504 | 32.0 (26.7–38)  30.4 (25.7-35.5) | *0.5904* | - | - | *-* |
| **Treatment line**  Non-first  First | 178/672  105/238 | 26.5 (22.7–30.7)  44.1 (36.1-53.4) | *<0.0001* | 0.6939 | 0.1860 | *0.0002* |
| **N° of metastatic sites**  >2  ≤ 2 | 119/465  164/445 | 25.6 (17.8–74.2)  36.9 (29.3-39.9) | *0.0002* | 0.3731 | 0.1568 | *0.0173* |
| **ECOG PS**  ≥2  0-1 | 20/126  263/784 | 15.9 (9.7–24.5)  33.5 (29.6-37.8) | *0.0001* | 0.6915 | 0.2689 | *0.0010* |
|  | | | | **Nagelkerke R2: 0.1629** | | |
